# Supplementary material for: Electrochemically coupled CH4 and CO2 consumption driven by microbial processes
Source: Nat Commun. 2024 Apr 10;15:3097. doi: 10.1038/s41467-024-47445-8 (PMC11006836; doi:10.1038/s41467-024-47445-8)
Supplement: Supplementary file 2 — Description of Additional Supplementary Files [file 41467_2024_47445_MOESM2_ESM.pdf]

Description of Additional supplementary file

Supplementary data 1: Global distribution of Methylomonas, Bacillus and Rhodopseudomonas. The Methylomonas, Bacillus and Rhodopseudomonas were identified based on the database of Microbiome Search Engine 2 (MSE2, http://mse.ac.cn).
