# Supplementary material for: Electrochemically coupled CH4 and CO2 consumption driven by microbial processes
Source: Nat Commun. 2024 Apr 10;15:3097. doi: 10.1038/s41467-024-47445-8 (PMC11006836; doi:10.1038/s41467-024-47445-8)
Supplement: Supplementary file 3 — Supplementary Information [file 41467_2024_47445_MOESM3_ESM.pdf]

Supporting Information

Electrochemically coupled CH_4 and CO_2 consumption driven by microbial processes

Yue Zheng[1,2,†], Huan Wang[1,2,†], Yan Liu[3,4], Peiyu Liu[3,4], Baoli Zhu[5], Yanning Zheng[6], Jinhua Li[3,4], Ludmila Chistoserdova[7], Zhiyong Jason Ren[8,*], Feng Zhao[1,*]

[1] CAS Key Laboratory of Urban Pollutant Conversion, Institute of Urban Environment, Chinese Academy of Sciences, Xiamen 361021, China.

[2] State Key Laboratory of Marine Environmental Science, and College of the Environment and Ecology, Xiamen University, Xiamen, 361102, China.

[3] Key Laboratory of Earth and Planetary Physics, Institute of Geology and Geophysics, Chinese Academy of Sciences, Beijing 100029, China.

[4] Laboratory for Marine Geology, Qingdao National Laboratory for Marine Science and Technology, Qingdao 266061, China.

[5] Key Laboratory of Agro-ecological Processes in Subtropical Regions and Taoyuan Agro-ecosystem Research Station, Institute of Subtropical Agriculture, Chinese Academy of Sciences, Changsha 410125, China.

[6] State Key Laboratory of Microbial Resources, Institute of Microbiology, Chinese Academy of Sciences, Beijing 100101, China.

[7] Department of Chemical Engineering, University of Washington, Seattle, USA.

8 Department of Civil and Environmental Engineering, and Andlinger Center for Energy and the Environment, Princeton University, 41 Olden St. Princeton, NJ 08540, USA.

† These authors contributed equally to this work.

* Corresponding authors: Zhiyong Jason Ren (zjren@princeton.edu) and Feng Zhao (fzhao@iue.ac.cn).

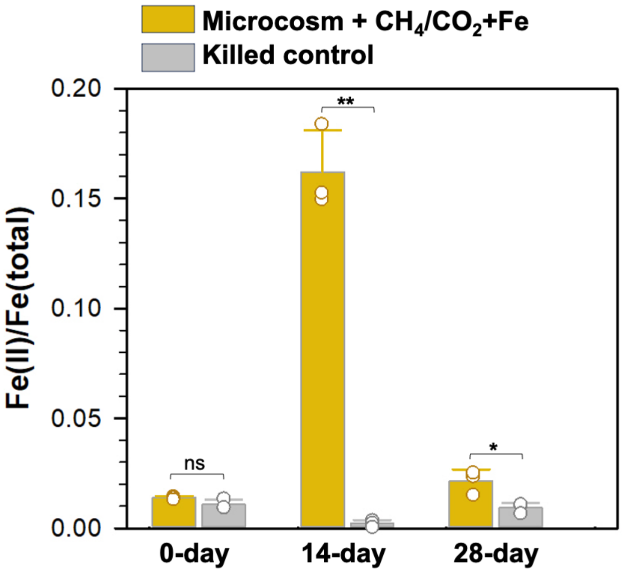

Supplementary Figure 1. The change of Fe(II)/Fe(total) in the microcosmic experiment. The Fe(II)/Fe(total) ratio was measured at three different time points: 0-day (initial phase), 14-day (reduced mineral phase), and 28-day (oxidized mineral phase). The killed control represents the incubation with sterilized soil in microcosm systems. Data generated from n = 3 biologically independent samples for each group and error bars indicate standard deviation of the mean. Asterisks indicate the range of *P* values calculated from two-tailed t-test paring (*, $P <= 0.05$; **, $P <= 0.01$; *ns*, $P > 0.05$).

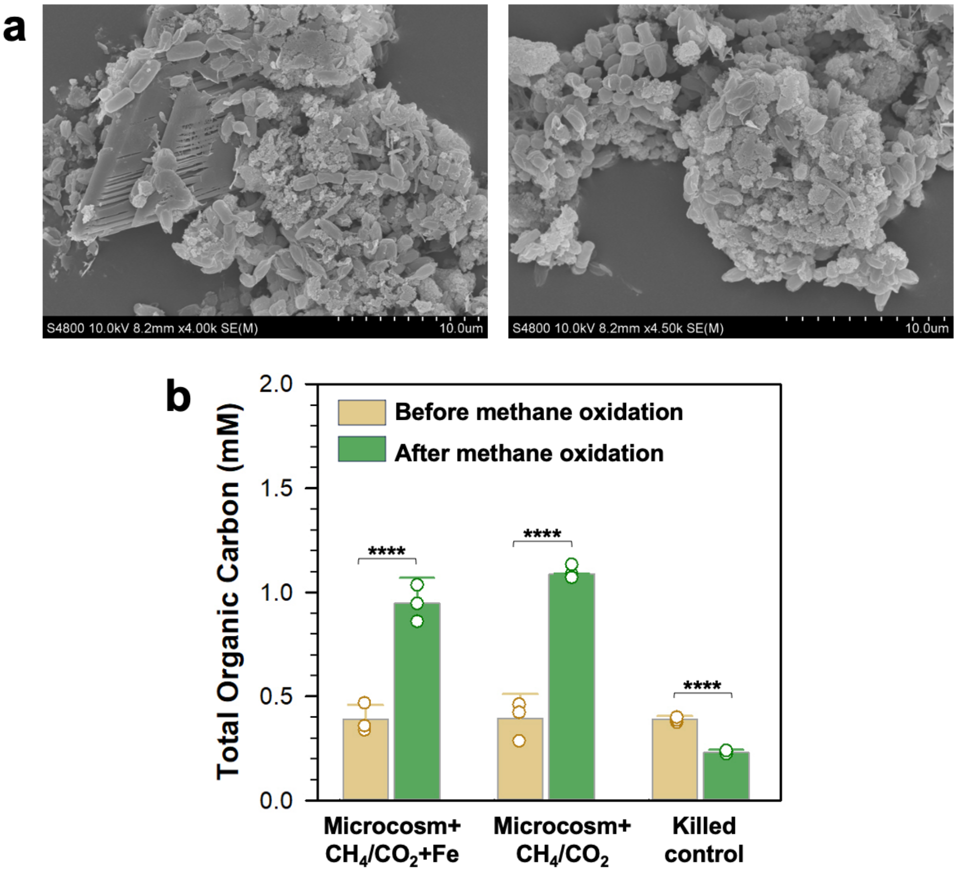

Supplementary Figure 2. **The images of microorganisms-minerals aggregates and the organic metabolites produced in microcosm systems.** (a) Microbial aggregates on the surface of the iron mineral by scanning electron microscopy; representative of 12 images. (b) The production of total organic carbon before and after methane oxidation in microcosm systems with or without iron minerals. The killed control represents the incubation with sterilized soil in microcosm systems. Data generated from n = 3 biologically independent samples for each group and error bars indicate standard deviation of the mean. Asterisks indicate the range of P values calculated from two-tailed t-test paring (****, $P <= 0.0001$).

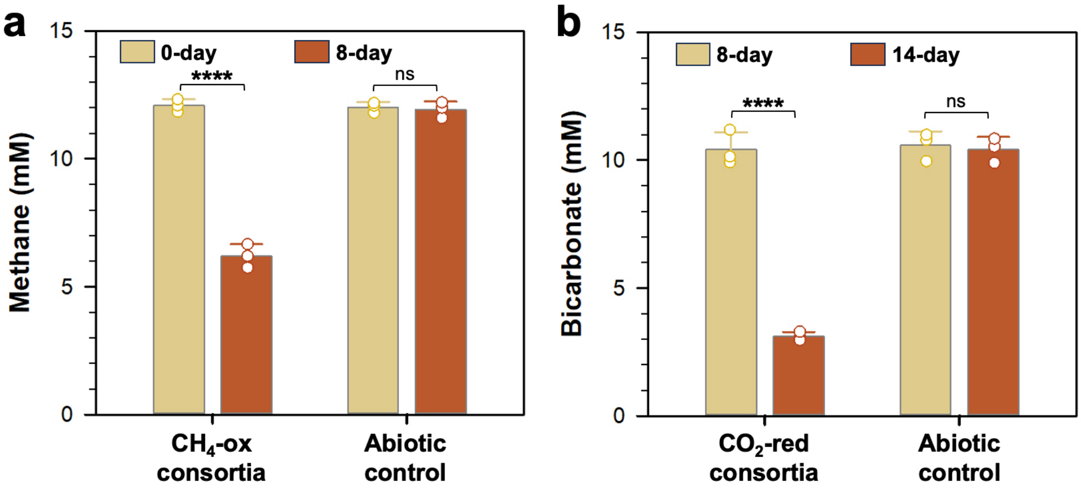

Supplementary Figure 3. The change of concentrations of (a) methane and (b) bicarbonate in the experiments of enriched microbial consortia. The concentration of methane was measured at two different time points: 0-day (initial phase) and 8-day (reduced mineral phase). The concentration of bicarbonate was measured at two different time points: 8-day (reduced mineral phase) and 14-day (oxidized mineral phase). The abiotic control represents the incubation without microbial cells. Data generated from n = 3 biologically independent samples for each group and error bars indicate standard deviation of the mean. Asterisks indicate the range of *P* values calculated from two-tailed t-test paring (****, $P <= 0.0001$; *ns*, $P > 0.05$).

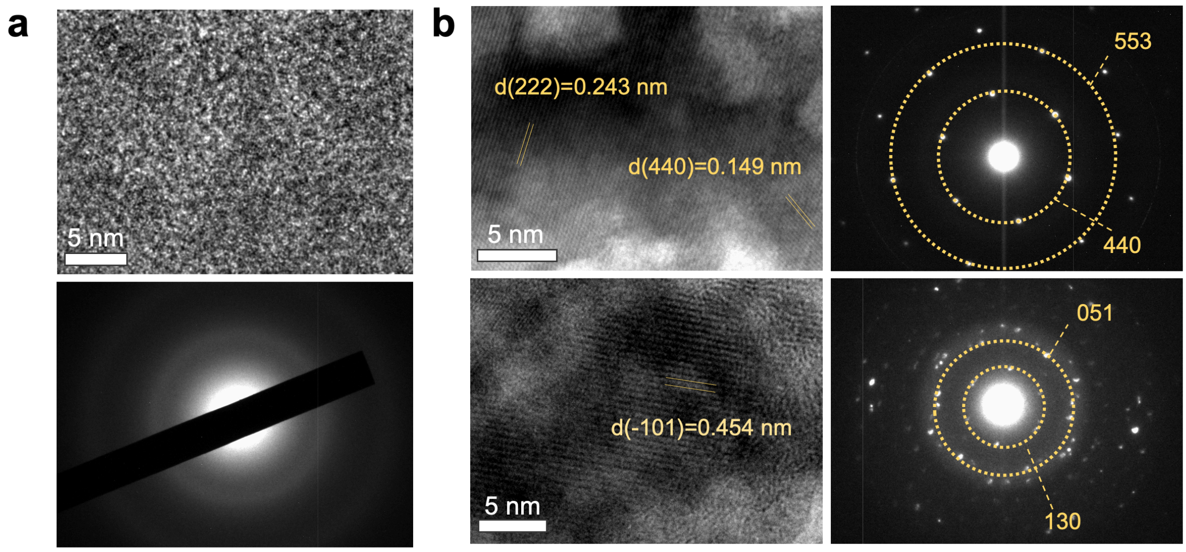

Supplementary Figure 4. **The lattice structure of iron mineral before iron reduction and after iron oxidation.** (a) Selected area electron diffraction and lattice images of iron mineral samples collected at Fe_{intial} time points as shown in Figure 2a. (b) Selected area electron diffraction and lattice images of iron mineral samples collected at Fe_{ox} time points as shown in Figure 2a.

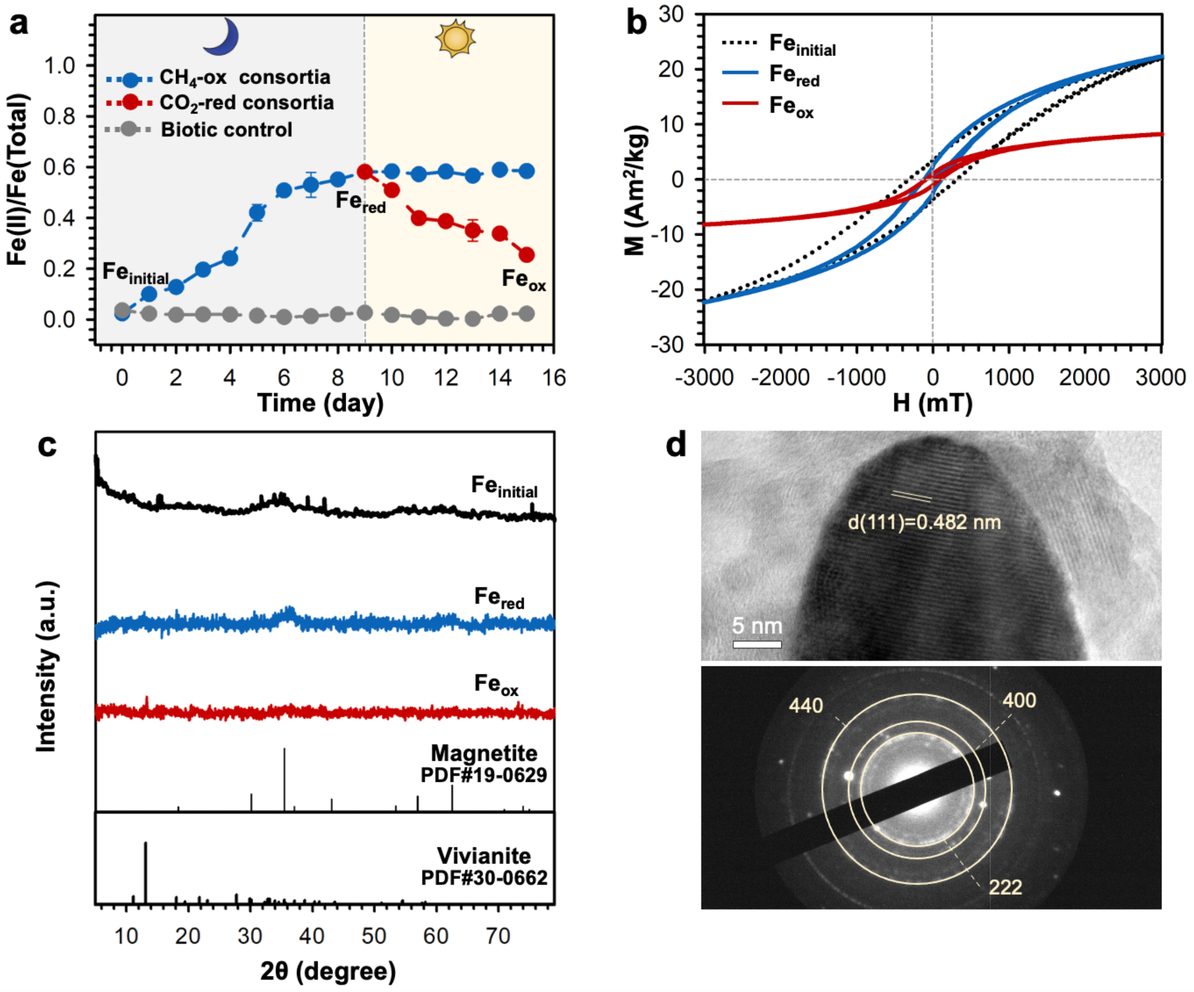

Supplementary Figure 5. Mineral characteristic of iron minerals without phosphate before and after reduction/oxidation. (a) The redox cycle of iron minerals was switched by light/dark conditions. The blue line means the participation of CH_4-oxidizing consortia (CH_4-ox consortia), and the red line means the participation of CO_2-reducing consortia (CO_2-red consortia). The abiotic control represents the incubation without microbial cells, and the biotic control represents the incubation without CH_4 and CO_2. Fe_{intial}, Fe_{red}, and Fe_{ox} represent the initial ferrihydrite, reduced iron minerals, and oxidized iron minerals. Data generated from n = 3 biologically independent samples for each group and error bars indicate standard deviation of the mean. (b) Magnetic hysteresis curves of three samples from $Fe_{initial}$, Fe_{red}, and Fe_{ox}. (c) X-ray diffraction of three samples from $Fe_{initial}$, Fe_{red}, and Fe_{ox}. (d) Selected area electron diffraction and lattice images of samples collected at Fe_{red}.

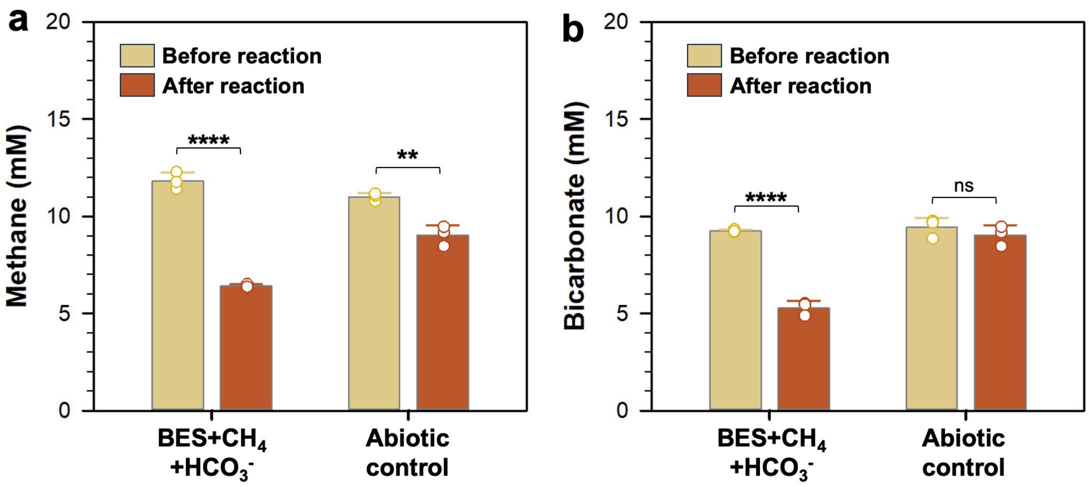

Supplementary Figure 6. The anodic CH_4 consumption and cathodic bicarbonate consumption in the two-chamber bioelectrochemical systems. The change of concentrations of (a) methane and (b) bicarbonate in anode chamber and cathode chamber of bioelectrochemical systems, respectively. Abiotic control represents corresponding control that is without microbial cells. Data generated from n = 3 biologically independent samples for each group and error bars indicate standard deviation of the mean. Asterisks indicate the range of P values calculated from two-tailed t-test paring (****, $P <= 0.0001$; **, $P <= 0.01$; *ns*, $P > 0.05$).

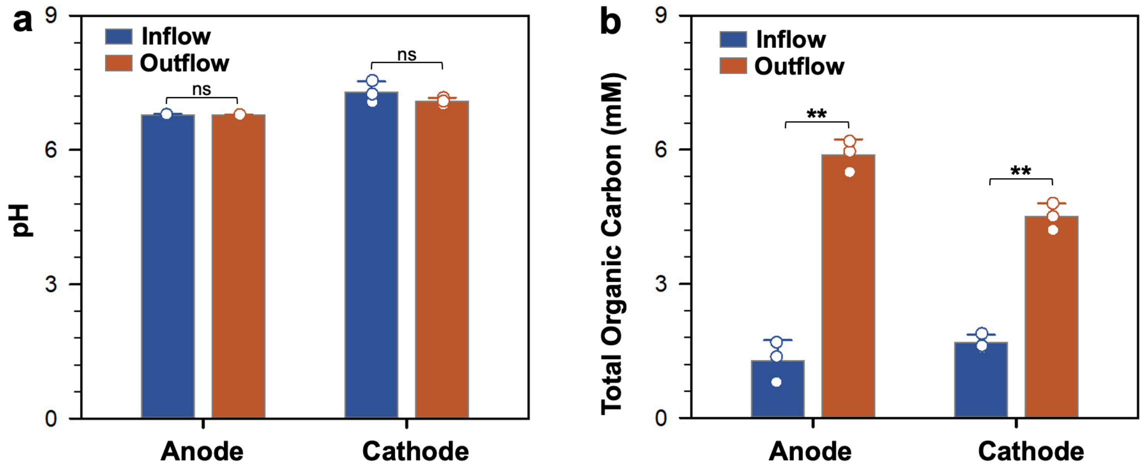

Supplementary Figure 7. The change of pH and total organic carbon of the two-chamber bioelectrochemical system. (a) The change of pH of the anode chamber and cathode chamber of the two-chamber bioelectrochemical system. (b) The change of total organic carbon of the anode chamber and cathode chamber of the two-chamber bioelectrochemical system. Data generated from n = 3 biologically independent samples for each group and error bars indicate standard deviation of the mean. Asterisks indicate the range of P values calculated from two-tailed t-test paring (**, $P <= 0.01$; *ns*, $P >0.05$).

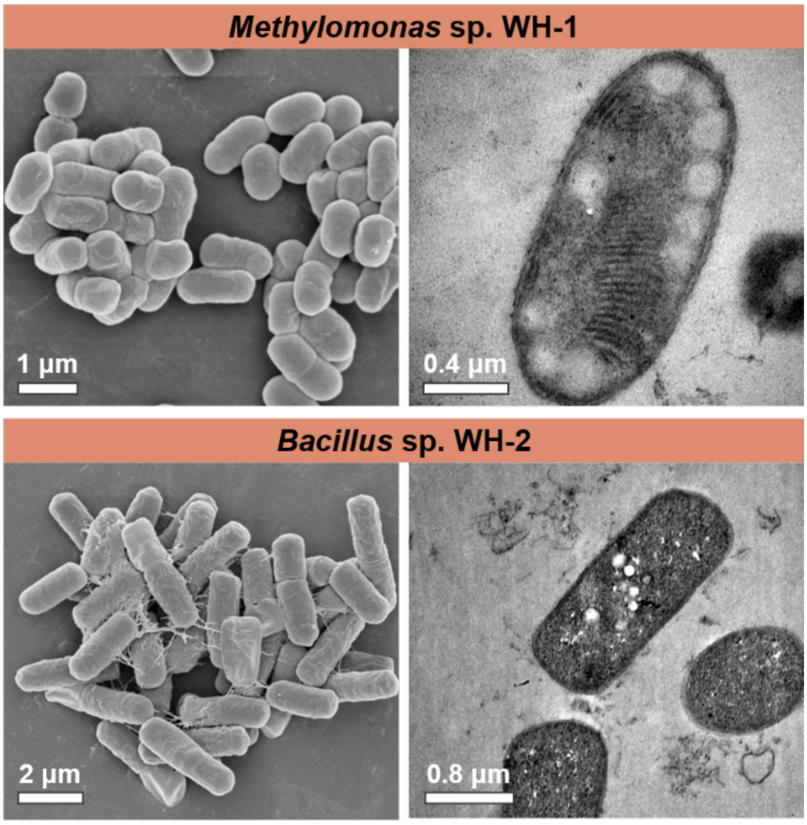

Supplementary Figure 8. The images of scan electron microscopy (SEM) and transmission electron microscopy (TEM) of *Methylomonas* sp. WH-1 and *Bacillus* sp. WH-2; representative of 10 images.

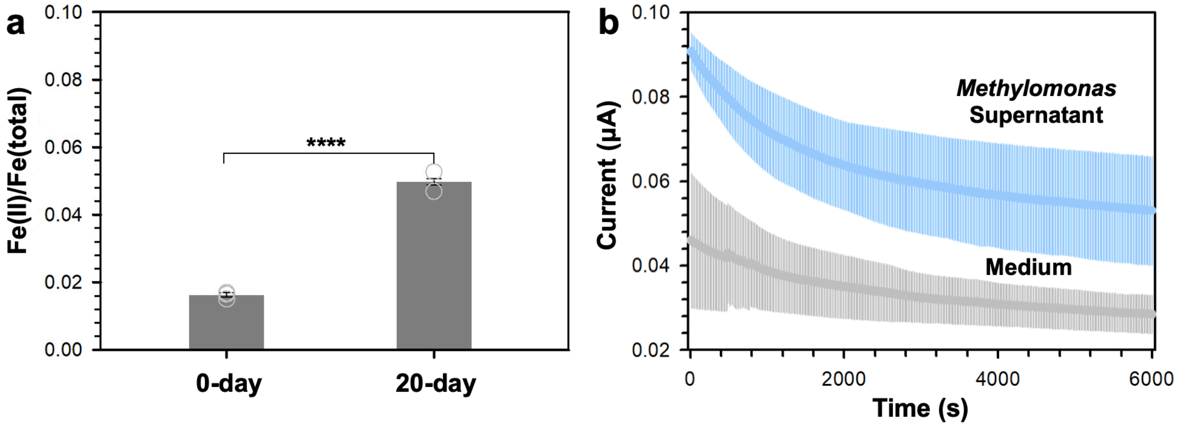

Supplementary Figure 9. The performances of iron reduction and electric current production of *Bacillus* sp. WH-2 in the supernatant of *Methylomonas* sp. WH-1. (a) Iron reduction of *Bacillus* sp. WH-2 in the supernatant of *Methylomonas* sp. WH-1 under hypoxic conditions. (b) Electrochemical response of *Bacillus* sp. WH-2 in the supernatant of *Methylomonas* sp. WH-1 as electrolyte at the potential of 0.3 V (vs. Ag/AgCl). The AMS medium as electrolyte was used as a control. Data generated from n = 3 biologically independent samples for each group and error bars indicate standard deviation of the mean. Asterisks indicate the range of *P* values calculated from two-tailed t-test paring (****, $P <= 0.0001$).

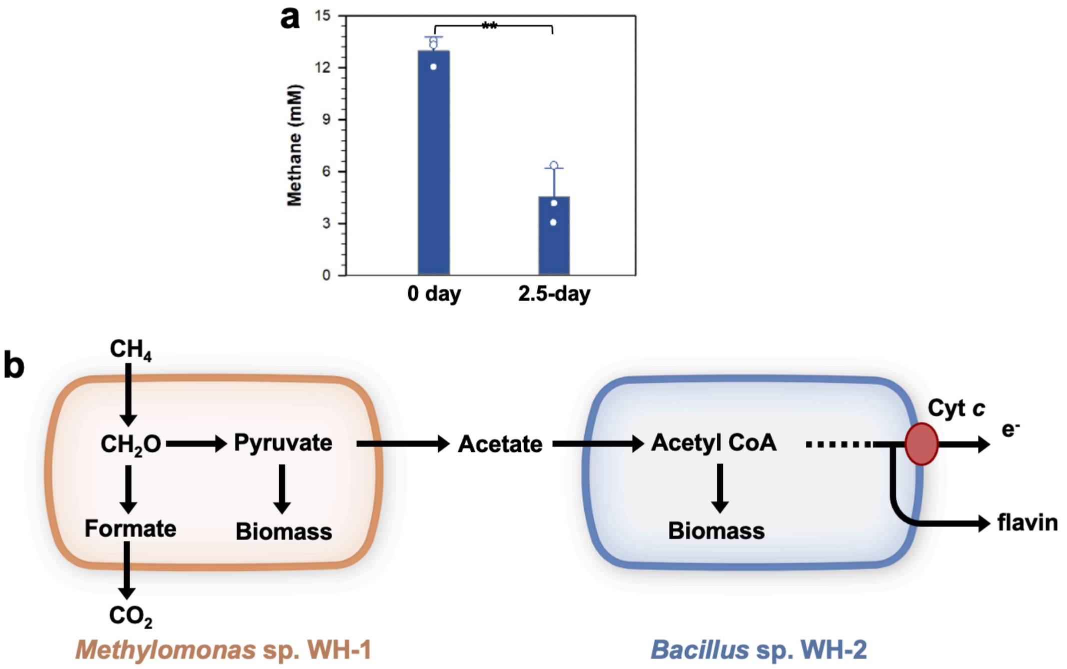

Supplementary Figure 10. The methane consumption and potential metabolic interactions of the co-culture in anode chamber. (a) The change of concentrations of methane of the co-culture of *Methylomonas* sp. WH-1 and *Bacillus* sp. WH-2 in the anode chamber. Data generated from n = 3 biologically independent samples for each group and error bars indicate standard deviation of the mean. Asterisks indicate the range of P values calculated from two-tailed t-test paring (**, $P <= 0.01$). (b) The conceptual schematic of the cross-feeding between *Methylomonas* sp. WH-1 and *Bacillus* sp. WH-2 that agrees with the measurements and the genome annotation.

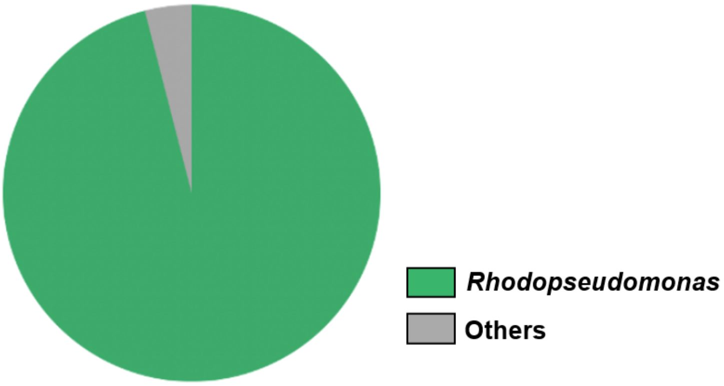

Supplementary Figure 11. The microbial composition in the three-electrode bioelectrochemical systems of CO_2-reducing consortia. Data generated from n = 3 biologically independent samples for each group.

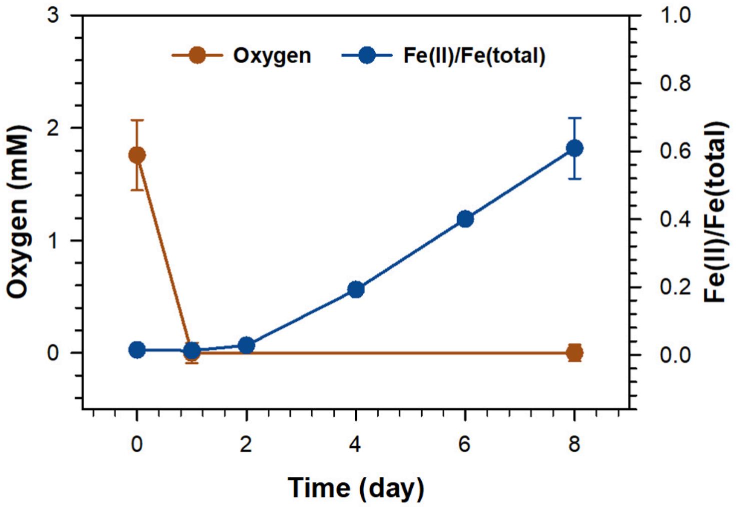

Supplementary Figure 12. The change of the concentration of oxygen and the ratio of Fe(II)/Fe(total) during shift from aerobic to anaerobic condition. Data generated from n = 4 biologically independent samples for each group and error bars indicate standard deviation of the mean.

Supplementary Table 1. Mineral characteristics of three samples from Fe_{intial}, Fe_{red}, and Fe_{ox}. saturation remanence (*Mrs*), Saturation magnetization (*Ms*), Near-zero coercivity (*Hc*).

Sample	*Mrs* (Am^2/kg)	*Ms* (Am^2/kg)	*Hc* (mT)
$Fe_{initial}$	3.45	22.00	321.37
Fe_{red}	0.75	29.31	40.21
Fe_{ox}	0.03	17.12	5.05
