# Supplementary material for: Electrochemically coupled CH4 and CO2 consumption driven by microbial processes
Source: Nat Commun. 2024 Apr 10;15:3097. doi: 10.1038/s41467-024-47445-8 (PMC11006836; doi:10.1038/s41467-024-47445-8)
Supplement: Supplementary file 5 — Reporting Summary [file 41467_2024_47445_MOESM5_ESM.pdf]

nature portfolio

Corresponding author(s): Zhiyong Jason Ren, Feng Zhao

Last updated by author(s): Mar 15, 2024

# Reporting Summary

Nature Portfolio wishes to improve the reproducibility of the work that we publish. This form provides structure for consistency and transparency in reporting. For further information on Nature Portfolio policies, see our Editorial Policies and the Editorial Policy Checklist.

## Statistics

For all statistical analyses, confirm that the following items are present in the figure legend, table legend, main text, or Methods section.

| n/a | Confirmed | |
|---|---|---|
| ☐ | ☒ | The exact sample size (*n*) for each experimental group/condition, given as a discrete number and unit of measurement |
| ☐ | ☒ | A statement on whether measurements were taken from distinct samples or whether the same sample was measured repeatedly |
| ☐ | ☒ | The statistical test(s) used AND whether they are one- or two-sided<br>*Only common tests should be described solely by name; describe more complex techniques in the Methods section.* |
| ☒ | ☐ | A description of all covariates tested |
| ☒ | ☐ | A description of any assumptions or corrections, such as tests of normality and adjustment for multiple comparisons |
| ☐ | ☒ | A full description of the statistical parameters including central tendency (e.g. means) or other basic estimates (e.g. regression coefficient) AND variation (e.g. standard deviation) or associated estimates of uncertainty (e.g. confidence intervals) |
| ☐ | ☒ | For null hypothesis testing, the test statistic (e.g. *F*, *t*, *r*) with confidence intervals, effect sizes, degrees of freedom and *P* value noted<br>*Give P values as exact values whenever suitable.* |
| ☒ | ☐ | For Bayesian analysis, information on the choice of priors and Markov chain Monte Carlo settings |
| ☒ | ☐ | For hierarchical and complex designs, identification of the appropriate level for tests and full reporting of outcomes |
| ☒ | ☐ | Estimates of effect sizes (e.g. Cohen's *d*, Pearson's *r*), indicating how they were calculated |

*Our web collection on statistics for biologists contains articles on many of the points above.*

## Software and code

Policy information about availability of computer code

| | |
|---|---|
| Data collection | No software was used. |
| Data analysis | Software used for this project include. Genomic analysis: NGsacToolkit v2.3, fastUnig v1.1, BLESS v1.01, Edena v3.131028. Microbial community analysis: QllME v2023.2, Trimmomatic v0.39. |

For manuscripts utilizing custom algorithms or software that are central to the research but not yet described in published literature, software must be made available to editors and reviewers. We strongly encourage code deposition in a community repository (e.g. GitHub). See the Nature Portfolio guidelines for submitting code & software for further information.

## Data

Policy information about availability of data

All manuscripts must include a data availability statement. This statement should provide the following information, where applicable:

- Accession codes, unique identifiers, or web links for publicly available datasets
- A description of any restrictions on data availability
- For clinical datasets or third party data, please ensure that the statement adheres to our policy

The complete data of this study and a complete data availability statement are provided in the manuscript under the section of 'Data availability'.

## Research involving human participants, their data, or biological material

Policy information about studies with human participants or human data. See also policy information about sex, gender (identity/presentation), and sexual orientation and race, ethnicity and racism.

| | |
|---|---|
| Reporting on sex and gender | N/A |
| Reporting on race, ethnicity, or other socially relevant groupings | N/A |
| Population characteristics | N/A |
| Recruitment | N/A |
| Ethics oversight | N/A |

Note that full information on the approval of the study protocol must also be provided in the manuscript.

# Field-specific reporting

Please select the one below that is the best fit for your research. If you are not sure, read the appropriate sections before making your selection.

☐ Life sciences ☐ Behavioural & social sciences ☒ Ecological, evolutionary & environmental sciences

For a reference copy of the document with all sections, see nature.com/documents/nr-reporting-summary-flat.pdf

# Ecological, evolutionary & environmental sciences study design

All studies must disclose on these points even when the disclosure is negative.

| | |
|---|---|
| Study description | Our study demonstrates the coupling of methane oxidation and carbon dioxide reduction in a switched biological process regulated by the redox cycling of iron minerals. By using both microcosm and enrichment cultures. We found iron minerals can act as an energy bridge between the methane-oxidizing and the carbon dioxide-reducing consortia that enables concurrent fixations of both greenhouse gases. The iron minerals acted as the electron acceptor for methane oxidation and the electron donor for carbon dioxide reduction, which are facilitated by changes in the mineral structure. All experiments were replicated a minimum of three times independently, with consistent results. |
| Research sample | The soil used in this study was sampled in Xiamen, which is located at 24°45'22'' N and 118°4'2'' W. The sample used in this study was from a paddy soil which is typical of the hotspots for greenhouse gas emissions. Paddy soil is one of the main habitats for methanotrophic bacteria, commonly used for enriching methanotroph and other electroactive bacteria. Thus, this work uses paddy soil as a inoculum to demonstrate the novel approach of strategic coupling of CH4 oxidation and CO2 reduction. |
| Sampling strategy | We collected soil from four corners of approximately a 4x4 m square and soil were taken from the water-soil interface by sterile equipment. The size of the sample was not precalculated. The samples were first resuspended in the culture medium, and then aliquoted into several culture flasks for microcosms or enriching consortia. The incubation was carried out on three replicates per treatment. |
| Data collection | Yue Zheng and Huan Wang carried out and performed the soil sampling and microcosm experiment. The XRD were measured by X'Pert Pro (PANalytical, Netherlands) carried by Huan Wang. The HTEM were observed on JEM-2100HR transmission electron microscope (JEM-2100HR, JEOL, Japan) and recorded by Yan Liu. Magnetic measurements were performed on MPMS XP-XL5 (Quantum Design, USA). For magnetic measurements, samples were filled into the non-magnetic capsule with a self-made non-magnetic spoon and the measurements were performed using MPMS XP-XL5 (Quantum Design, USA). DNA extraction was done by Huan Wang using the Qiagen PowerSoil kits. Amplicon sequencing data was generated using Illumina MiSeq and the data analysis was by Yue Zheng. The labeled carbon were measured using the Gas Source Isotopic Ratio Mass Spectrometer (IRMS, MAT253 PLUS, Thermo, USA), TOC analyzer (isoTOC cube, Elementar, Germany) coupled with IRMS (IsoPrime100, Elementar, UK), IRMS (Delta V Advantage, Thermo, USA) with a GasBench II Autosampler (CombiPAL, CTC Analytics, Switzerland), elemental analyzer (Flash EA 2000, Thermo, Germany) combined with IRMS (MAT253 Plus, Thermo, USA). The SEM and TEM imaging was done by Huan Wang on a SEM observation (S-4800 FE-SEM, Hitachi, Japan) and TEM (H-7650, Hitachi, Japan). The output voltage of BES was recorded by a digital multimeter (Keithley Instruments, USA). The volatile fatty acids were measured by ion chromatography (ICS-3000, Dionex, UAS). The chronoamperometry was performed on electrochemical workstation (CHI 832, Chenhua, China). |
| Timing and spatial scale | This study took place between 03/2020-09/2022. The key experiments were listed as folllows. The microcosm experiments and enrichment experiments were done from March 2020 to July 2022. The electrochemical studies including the construction of bioelectrochemical systems and the measurment of electrical current response were studied during 08/2020-06/2021. DNA extraction and sequencing was done in December 2020 and took approximately 20 days. Isotopic labelling experiments was done in July 2022 and lasted for one month. |
| Data exclusions | There was no sample was excluded. |

| | |
|---|---|
| Reproducibility | Each condition/group underwent at least three biological replicates and similar results were obtained. |
| Randomization | The soil sampling location was fully randomized. |
| Blinding | Every treatment was given unique ID numbers. During sample processing blinding was not possible because some replicates from the same treatments were grouped, thus group allocation had to be known. During parameter measuring, the experimental methods were applied to all samples. During data analyses, the data analysis scripts were applied to all samples simultaneously. |

Did the study involve field work? ☐ Yes ☒ No

# Reporting for specific materials, systems and methods

We require information from authors about some types of materials, experimental systems and methods used in many studies. Here, indicate whether each material, system or method listed is relevant to your study. If you are not sure if a list item applies to your research, read the appropriate section before selecting a response.

## Materials & experimental systems

| n/a | Involved in the study |
|---|---|
| ☒ | ☐ Antibodies |
| ☒ | ☐ Eukaryotic cell lines |
| ☒ | ☐ Palaeontology and archaeology |
| ☒ | ☐ Animals and other organisms |
| ☒ | ☐ Clinical data |
| ☒ | ☐ Dual use research of concern |
| ☒ | ☐ Plants |

## Methods

| n/a | Involved in the study |
|---|---|
| ☒ | ☐ ChIP-seq |
| ☒ | ☐ Flow cytometry |
| ☒ | ☐ MRI-based neuroimaging |

## Plants

| | |
|---|---|
| Seed stocks | N/A |
| Novel plant genotypes | N/A |
| Authentication | N/A |
